# Pentoxifylline Inhibits WNT Signalling in β-Catenin^high^ Patient-Derived Melanoma Cell Populations

**DOI:** 10.1371/journal.pone.0158275

**Published:** 2016-06-28

**Authors:** Beata Talar, Anna Gajos-Michniewicz, Marcin Talar, Salem Chouaib, Malgorzata Czyz

**Affiliations:** 1 Department of Molecular Biology of Cancer, Medical University of Lodz, Lodz, Poland; 2 Department of Haemostasis and Haemostatic Disorders, Medical University of Lodz, Lodz, Poland; 3 Unité INSERM U1186, Institut de Cancérologie Gustave Roussy, Villejuif, France; University of Colorado, School of Medicine, UNITED STATES

## Abstract

**Background:**

The heterogeneity of melanoma needs to be addressed and combination therapies seem to be necessary to overcome intrinsic and acquired resistance to newly developed immunotherapies and targeted therapies. Although the role of WNT/β-catenin pathway in melanoma was early demonstrated, its contribution to the lack of the melanoma patient response to treatment was only recently recognized. Using patient-derived melanoma cell populations, we investigated the influence of pentoxifylline on melanoma cells with either high or low expression of β-catenin.

**Findings:**

Our results indicate that pentoxifylline inhibits the activity of the canonical WNT pathway in melanoma cell populations with high basal activity of this signalling. This is supported by lowered overall activity of transcription factors TCF/LEF and reduced nuclear localisation of active β-catenin. Moreover, treatment of β-catenin^high^ melanoma cell populations with pentoxifylline induces downregulation of genes that are targets of the WNT/β-catenin pathway including connective tissue growth factor (CTGF) and microphthalmia-associated transcription factor (MITF-M), a melanocyte- and melanoma cell-specific regulator.

**Conclusions:**

These results suggest that pentoxifylline, a drug approved by the FDA in the treatment of peripheral arterial disease, might be tested in a subset of melanoma patients with elevated activity of β-catenin. This pharmaceutical might be tested as an adjuvant drug in combination therapies when the response to immunotherapy is prevented by high activity of the WNT/β-catenin pathway.

## Introduction

Melanoma is one of the most lethal cancers. Despite extensive research, therapeutic options for patients with advanced melanoma remain unsatisfactory, and acquired resistance to treatment is observed in the majority of them. The results of targeted therapies and immunotherapies suggest that combination treatment might be helpful to overcome intrinsic and acquired resistance [1–6]. Most recently, it has been reported that the Wingless (WNT)/β-catenin signalling pathway prevents anti-melanoma immune responses [7]. The WNT/β-catenin pathway plays a crucial role in embryogenesis, stem cell maintenance and initiation and progression of many cancers [8]. It influences neural crest stem cell fate leading to the development of melanocytes [9]. The role of WNT/β-catenin signalling in melanoma seems to be more complex than in other cancers [10,11], and its cell-type specific role is partially mediated by MITF-M, a melanocyte- and melanoma-specific transcription factor [12].

FDA-approved pentoxifylline (BL-191, Trental) is clinically used in patients with chronic peripheral arterial disease to increase blood flow and enhance tissue oxygenation. Several activities of pentoxifylline were recognized in preclinical studies both *in vitro* and *in vivo*. Pentoxifylline was shown to inhibit TNF-α production [13] and MMP-2 and MMP-9 secretion [14], induce apoptosis related to upregulation of DR4 and DR5 (death receptor-4 and -5) expression on the cell surface and downregulation of anti-apoptotic proteins [15]. Pentoxifylline was demonstrated to be involved in downregulation of the PKC, MAPK and induction of NF-κB signalling in vascular smooth muscle cells [16]. In melanoma cell lines, pentoxifylline combined with irradiation significantly increased radiotoxicity in a *TP53* mutant cell line and effectively suppressed DNA double-strand break repair [17], inhibited the G_1_-S phase transition [14], and increased the activity of glutathione-S-transferase leading to glutathione depletion [18]. Most recently, it was demonstrated that pentoxifylline induced ER stress response and autophagy in melanoma cells [19]. In *in vivo* experiments, it significantly inhibited subcutaneous melanoma xenograft growth and angiogenesis without any toxicity [20,21].

In our previous study, we have provided evidence indicating that pentoxifylline efficiently reduced percentages of cells with clonogenic potential but was less effective in decreasing overall cell viability [22]. We have also demonstrated that pentoxifylline markedly reduced the frequency of ABCB5 (ATP-binding cassette, sub-family B, member 5)-positive cells that are considered as melanoma-initiating cells [23]. Thus, our previous results position pentoxifylline as a drug targeting melanoma stem-like cells [22]. Following the concept linking the cancer stemness with the WNT/β-catenin pathway [24], we asked whether pentoxifylline could affect the activity of the WNT/β-catenin pathway in melanoma. The diverse response to specific drugs is frequently observed in melanoma cell lines that differ in phenotypes [22,25,26]. Thus, for the present study, we have selected patient-derived melanoma populations with different expression of β-catenin, a crucial effector of the WNT/β-catenin signalling pathway.

## Materials and Methods

### Drug

Pentoxyfilline was purchased from Sigma-Aldrich (P1784). 100 mM stock solution for each experiment was prepared in Dulbecco’s Modified Eagle’s Medium (DMEM)/F12 (Lonza, BE12-719F).

### Cell cultures and ethics statement

DMBC11, 12, 17, 19, 21 cell populations were obtained in the Department of Molecular Biology of Cancer from surgical specimens of melanoma in advanced stages as previously described [27]. The histopathological characteristics of melanoma used to obtain melanoma cell populations was described previously [27,28]. The DMBC21 cell population was derived from melanoma classified as pT4bN1bM0. The study was approved by the Ethical Commission of the Medical University of Lodz and written informed consent was obtained from each patient. Cells were maintained in stem cells medium (SCM) consisting of DMEM/F12 (Lonza, BE12-719F), B-27 supplement (Gibco, A1895601), growth factors (10 ng/ml bFGF and 20 ng/ml EGF; 354060 and 354052 BD Biosciences,), insulin (10 mg/ml), heparin (1 ng/ml), antibiotics (100 IU/ml penicillin, 100 mg/ml streptomycin). The medium was exchanged twice a week.

### Viability Assay and doubling time

Drug-induced changes in cell viability after 24 h, 48 h and 72 h treatment were assessed by flow cytometry after propidium iodide (PI) staining (Sigma-Aldrich, 81845). To assess relative changes in the viable cell number, an automated cell viability analyzer was used according to standard procedures. Both parameters were analysed using a FACSVerse flow cytometer and FACSuite software (Becton Dickinson). The software program Graph Pad Prism was used to plot viability curves. Doubling time (DT) was calculated using the formula: DT = (*t—t*_*0*_)log2 / (*logA—logA*_*0*_), in which *t* and *t*_*0*_ are the times at which the cells were assessed and *A* and *A*_*0*_ are the absorbance at times *t* and *t*_*0*_, respectively.

### Cell Cycle Analysis

Melanoma cells were treated with pentoxifylline at 10 mM for 24 h. After incubation cells were collected and fixed with 70% (w/v) ethanol at -20°C. Cells were washed twice with PBS and resuspended in PI Staining Buffer containing RNase (Becton Dickinson, 550825). Following incubation for 30 min at room temperature, cells were analysed using a FACSVerse flow cytometer. ModFit LT 3.0 software was used to calculate the percentages in each cell cycle phase.

### Flow Cytometric Analysis of Apoptosis

Detection of cell death was carried out by dual staining with Annexin V-FITC and PI (Roche Diagnostics, Manheim, Germany). Melanoma cells were seeded into 12-well plates and treated with 10 mM pentoxifylline for 24 h. After treatment, cells were collected, centrifuged at 400 x g for 5 min and stained with Annexin V-FITC and PI for 15 min at room temperature in the dark. 30,000 events were analyzed for each sample by flow cytometer FACSVerse (Becton Dickinson), and results were processed by using FACSuite software (Becton Dickinson).

### TCF/LEF reporter assay

Cell lines were transduced with Cignal TCF/LEF Reporter (GFP) or negative control (GFP reporter construct with a minimal promoter) (Qiagen, CCS-018G) by nucleofection (Lonza). Three hours after transfection, cells were treated with 5 mM and 10 mM Pen for 24 h. Cells were harvested, trypsinized and washed with PBS. Pellet was resuspended in PBS and incubated with 7-aminoactinomycin D (7-AAD) (eBioscience, 00-6993-50) for 10 min at 4°C. Acquisition of 30,000 cells was performed using FACSVerse flow cytometer and data were analysed with FACSuite software.

### Immunophenotyping by Flow Cytometry

Melanoma cells were treated with pentoxifylline at 10 mM for 24 h. After fixation with 4% paraformaldehyde (20 min) and permeabilisation with 0,2% Triton X-100 (10 min), cells were incubated in PBS with 4% FBS to block non-specific protein-protein interactions, and then with appropriate antibodies. The following antibodies were used: anti-MITF, N-terminal (1:50, rabbit polyclonal, Abcam, ab170466) along with APC-conjugated goat anti-rabbit (1:400, Santa Cruz Biotechnology, sc-3846) and anti-Melan-A (4.8 μg/ml, mouse monoclonal, DAKO, M7196) along with FITC-conjugated goat anti-mouse (1:500, goat anti-mouse, BD Pharmingen, 555988). Typically, 30,000 cells/sample were analysed. Appropriate isotype controls were used. To exclude dead cells from the analysis, LIVE/DEAD^®^ Fixable Violet Dead Cell Stain Kit (Invitrogen, L34957) was used. Acquisition was performed using FACSVerse flow cytometer and analysed using BD FACSuite software.

### Fluorescence microscopy

Cells were grown Lab-Tek^™^ II Chamber Slide^™^ System (Thermo Scientific^™^ Nunc^™^) and fixed with 4% paraformaldehyde in PBS for 20 min and permeabilised for 10 min in 0.2% Triton X-100-PBS. For antigen retrieval, cells were blocked in 5% FBS/PBS at room temperature for 30 min. Then, cells were incubated for 2 h with primary antibody specific for active form of β-catenin dephosphorylated on Ser37 or Thr41 (1:100, mouse monoclonal, Millipore, 05–665). After washing cells were incubated with secondary antibody (1:500, donkey anti-rabbit, Alexa Fluor^®^ 555-conjugated, Abcam, ab150074) for 1 h, then washed with PBS three times. Polystyrene media chamber was removed and coverslip was mounted using ProLong^®^ Gold Anti-fade with DAPI (Life Technology, P-36931). Images were recorded using a Axio Examiner A1; Carl Zeiss epifluorescence microscope with CCD camera (Rolera EM-C^2^,Q-Imaging). The analysis of the immunofluorescence data were performed on images that were acquired using the following parameters: magnification 400x, exposure time for 15 ms (DAPI) and for 20 ms (Alexa Flour 555). It was conducted with ZEN microscope software (Carl Zeiss). Localisation of β-catenin in nucleus was performed as combined display of two channels in colour overlay mode. The pixel of red channel (Alexa Fluor^®^ 555) was plotted against blue channel (DAPI) in the diagram and each pixel pair with the same X/Y coordinates was displayed as a point. Regions in which both fluorescent dyes (DAPI and Alexa flour 555) were co-localised were displayed in a rose colour. Histograms of Alexa flour 555 fluorescence intensities (co-localised with DAPI) have been shown to demonstrate decreased intensity of fluorescence of β-catenin after Pen treatment.

### RNA isolation, cDNA synthesis, quantitative Real-Time PCR

Total RNA was collected and purified using Total RNA Isolation kit with mini column system (A&A Biotechnology,). The RNA concentration and purity was evaluated with a microplate reader (Infinite M200Pro, Tecan). Subsequently, 1 μg of total RNA was transcribed into cDNA using 300 ng of random primers and SuperScript II Reverse Transcriptase (Invitrogen Life Technologies, 18064–014). The evaluation of mRNA expression of selected genes was performed by quantitative real-time polymerase chain reaction (qRT-PCR) using the Rotor-Gene 3000 Real-Time DNA analysis system (Corbett Research). Amplification was performed using KAPA SYBR FAST qPCR Kit Universal 2X qPCR Master Mix (Kapa Biosystems, KK4602). Sequences of primers used in qRT-PCR were shown elsewhere [22,27], except for primers for TCF4 (Forward 5' CTG CCT TAG GGA CGG ACA AAG, Reverse 5' TGC CAA AGA AGT TGG TCC ATT TT) and LEF1 (Forward 5' AAT GAG AGC GAA TGT CGT TGC, Reverse 5' GCT GTC TTT CTT TCC GTG CTA). To calculate the relative expression of target genes *versus* a reference gene RPS17, a mathematical model including an efficiency correction for qRT-PCR was used.

### Western blot analysis

After 24h of incubation with pentoxifylline, melanoma cells were lysed using RIPA buffer containing freshly added protease and phosphatase inhibitors. Protein concentration was determined by Bradford assay (BioRad, 500–0006). Whole cell extracts were fractionated by 7% SDS-polyacrylamide gel transferred to a Immobilon-P PVDF membrane (Millipore). Then, the membrane was incubated for 1 h in blocking solution (5% non-fat milk in TBS-Tween 0.05%). Primary antibodies detecting active β-catenin (1:1000, mouse monoclonal, Millipore, 05–665), total β-catenin (1:1000, mouse monoclonal, Santa Cruz Biotechnology, sc-7963), MITF (1:1000, rabbit monoclonal, Cell Signaling, 12590) or β-actin (1:2500, rabbit polyclonal, Sigma-Aldrich, A2066) were used followed by binding the secondary HRP-conjugated anti-mouse or anti-rabbit antibodies (Santa Cruz Biotechnology, sc-2054, sc-2005). The proteins were visualized using Pierce ECL Western Blotting Substrate (Pierce, 32106).

### Enzyme-linked Immunosorbent Assay (ELISA) of DKK1

The ELISA kit Quantikine Human DKK1 Immunoassay (R&D Systems, DKK100) was used to determine the concentrations of DKK1 in the culture media. The optical density of each well was determined with a microplate reader (Infinite M200Pro). A standard curve and the concentrations of DKK1 in the samples were obtained by reducing the data using a four-parameter logistic curve fit.

### Data analysis

All statistical calculations were performed with GraphPad Prism software. Curve fitting was carried out using the non-linear regression with variable slope. Pairwise comparison (treated sample *vs* non-treated sample) were performed with Student’s t-test. Data are represented as the mean values ± SD. A value of *P < 0.05 was used to establish a significant difference between the mean values of tested parameters.

## Results

### Pentoxifylline decreases viability more efficiently in slow- than in fast-proliferating melanoma populations

**Fig 1:**
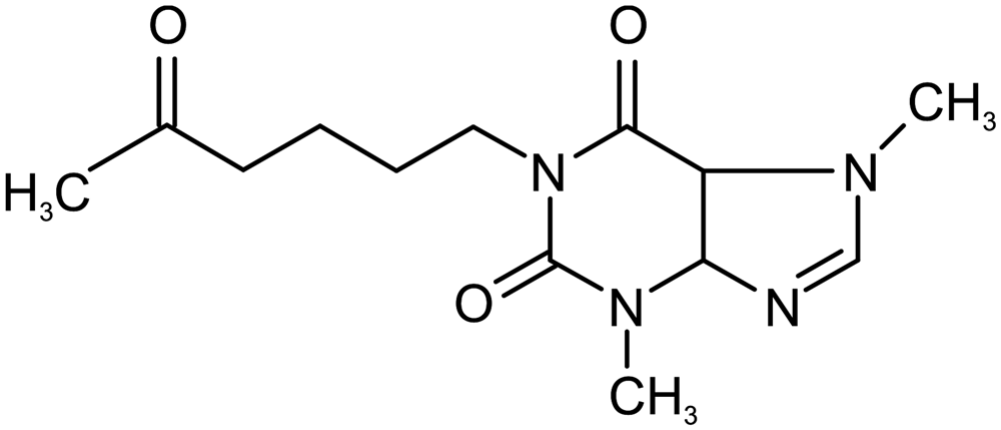
Chemical structure of pentoxifylline.

Stem cell medium (SCM) was used to propagate patient-derived melanoma cell cultures as we have previously shown that this medium containing growth factors EGF and bFGF better preserves the original tumour characteristics than serum-containing medium [29,30]. Five melanoma cell populations were used and three of them, DMBC12, DMBC11 and DMBC19 were fast-proliferating with doubling time 22 ± 2 h, 21 ± 3 h and 24 ± 3 h, respectively, whereas DMBC21 and DMBC17 were slow-cycling cell populations with a doubling time 62 ± 5 h and 54 ± 5 h, respectively. Four of the tested cell populations, DMBC11, DMBC12, DMBC19 and DMBC21, harbour mutation in BRAF (BRAF^V600E^), whereas DMBC17 cells are WT BRAF [31]. All these populations were treated with pentoxifylline (Fig 1), a drug that was previously selected from The Natural Products Set II [22].

**Fig 2:**
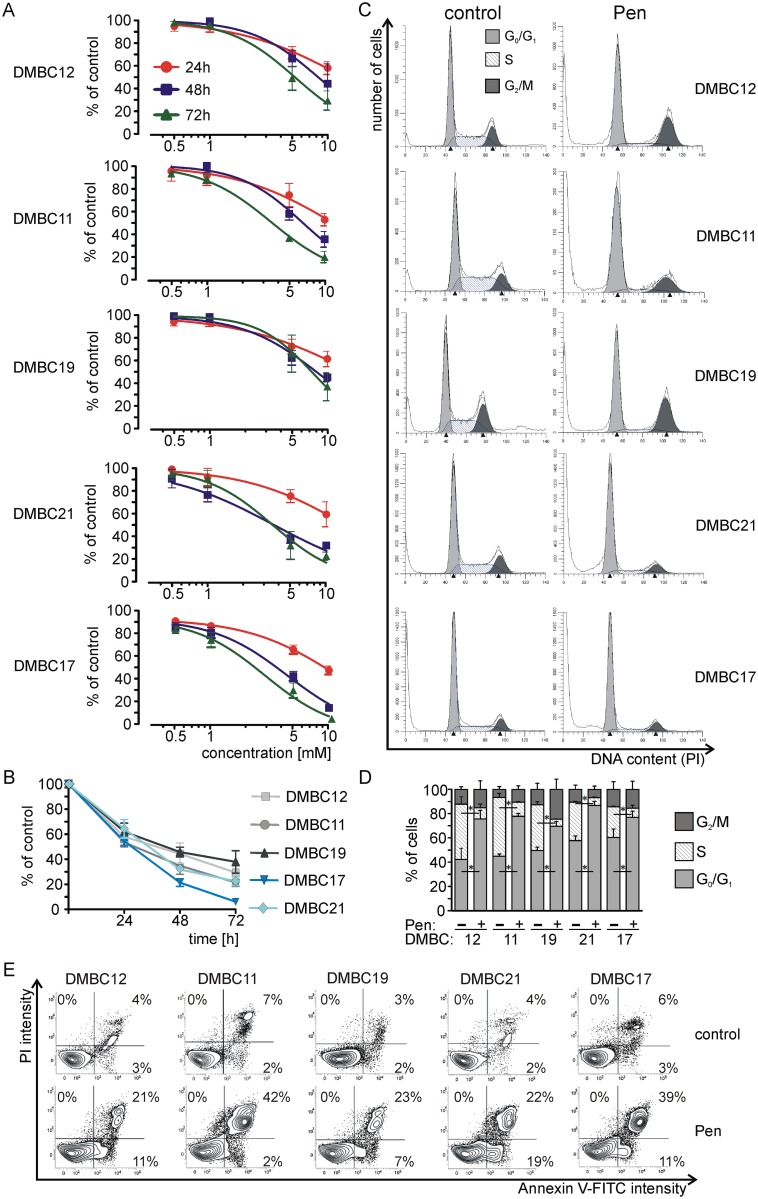
Pentoxifylline (Pen) reduces the viable cell number and induces cell cycle arrest in G_0_/G_1_ phase and apoptosis. **(A, B)** Concentration- and time-dependent effects of Pen on melanoma cell viability. DMBC12, DMBC11, DMBC19, DMBC21 and DMBC17 populations were treated with increasing concentrations of Pen (0.5–10 mM) for 24, 48 and 72 h. Changes in viability were estimated by flow cytometry after propidium iodide (PI) staining and expressed as the percentages of control. Data represent the mean ± SD of five independent experiments performed in triplicate. **(C, D)** Pen accumulates melanoma cells in G_0_/G_1_ phase. Distribution of melanoma cells in cell-cycle phases was determined by flow cytometry. Representative histograms **(C)** and their quantification from three independent experiments **(D)** are shown. ModFit LT 3.0 software was used to calculate the percentages of viable cells in cell-cycle phases. The differences between Pen-treated samples and respective control are considered significant at *P < 0.05. **(E)** Pen induces apoptosis in melanoma cells. Flow cytometry after Annexin V/propidium iodide staining was used to measure the percentages of melanoma cells in early and late apoptosis. Typical contour plots are shown.

Pentoxifylline reduced the relative number of viable cells in a concentration- and time-dependent manner (Fig 2A and 2B). A concentration-dependent decrease in viable cell number was analysed at various time points (Fig 2A). After 48 h and 72 h of treatment with pentoxifylline, the reduction of viable cell number was higher in slow-cycling populations, especially in DMBC17 than in populations with low doubling time. When the time-dependent effect of 10 mM pentoxifylline was assessed, again the DMBC17 population was the most sensitive one (Fig 2B). Cell cycle analysis was performed in melanoma cells exposed to pentoxifylline for 24 h (Fig 2C and 2D). Pentoxifylline-treated melanoma cells accumulated in G_0_/G_1_ phase, and this effect was significant in all melanoma populations.

Pentoxifylline at 10 mM which arrested melanoma cells mainly in G_0_/G_1_, induced apoptosis in less than 41% of cells within the first 24 h of treatment (Fig 2E). Again the DMBC17 cell population was the most sensitive one as the percentage of apoptotic cells (Annexin V-positive) was raised from 9% (control cells) to 50% (Pen-treated cells). The lowest effect was observed in the DMBC19 population, from 5% in the control to 30% in the pentoxifylline-treated cell population.

Concentrations lower than IC_50_, 5 mM or 10 mM, were then selected for a mechanistic study performed within the first 24 h of treatment. Of note, this concentration was cytotoxic and reduced viability of melanoma cells to 23% and 6% of control in DMBC21 and DMBC17 populations, respectively, when the incubation with pentoxifylline was prolonged to 72 h (Fig 2B).

### Pentoxifylline reduces the activity of WNT/β-catenin pathway

**Fig 3:**
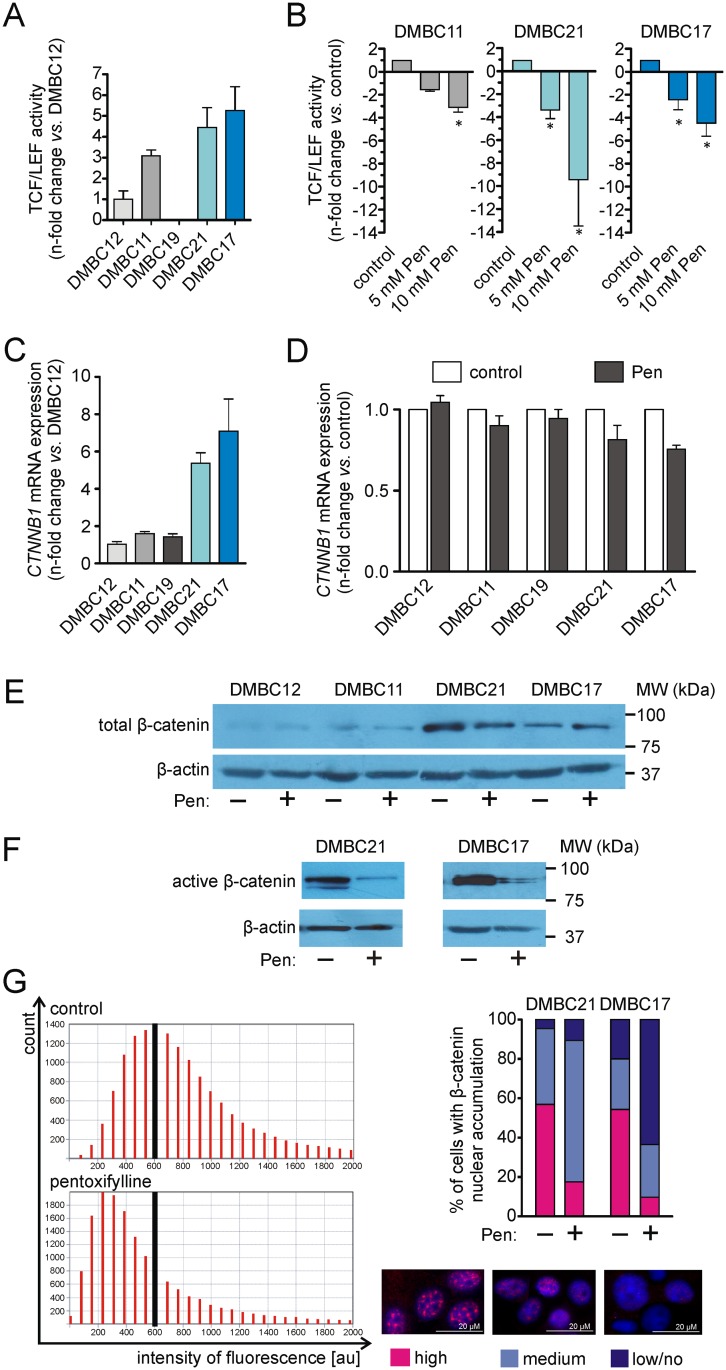
Pentoxifylline (Pen) downregulates WNT/β-catenin signalling assessed as activity of transcription factors TCF/LEF and the level of active β-catenin. **(A)** The basal TCF/LEF activity in DMBC11, DMBC12, DMBC17, DMBC19, DMBC21 populations determined 27 h after transfection with Cignal TCF/LEF fluorescent reporter (GFP). The activity in the DMBC12 population with the lowest detectable activity of TCF/LEF was used as the reference value. Data are presented as means ± SD. **(B)** The fold change in TCF/LEF activity after Pen treatment in cell populations with high TCF/LEF activity. Cells transfected with Cignal TCF/LEF Reporter (GFP) were treated with Pen at indicated concentrations for 24h. Data represent the fold change *vs*. control. The differences are considered significant at *P < 0.05. **(C)** qRT-PCR validation of *CTNNB1* expression in melanoma cell populations relative to its expression in the DMBC12 population. **(D)** *CTNNB1* mRNA fold change after 18 h incubation with 10 mM Pen. The expression level of *CTNNB1* was determined and normalized to the expression of a reference gene *RPS17*. Data are presented as fold change in Pen-treated samples *vs*. control. **(E, F)** Representative Western blot images showing the total **(E)** and active form **(F)** of β-catenin in melanoma cells. Cells were incubated with 10 mM Pen for 24 h. β-actin was used as a loading control. **(G)** Microscopic analysis of nuclear β-catenin in β-catenin^high^ melanoma cell populations. Presented histograms of β-catenin fluorescence intensity in nuclei of untreated cells and Pen-treated samples (left). The vertical black line on histograms demonstrates the shift in the fluorescence intensity observed for cells treated with Pen in comparison to control. Co-localisation of β-catenin and DAPI-stained nuclei in different cells was represented in the merge images (right). Counted cells (n = 300 in each group) depending on their fluorescence intensity was classified into to three categories: high, medium and low/none. Data are summarised as a bar graph.

To investigate the influence of pentoxifylline on the WNT/β-catenin pathway, changes in activity of transcription factors TCF/LEF were assessed using a fluorescent reporter assay. Tested populations differed in the basal TCF/LEF activity with almost undetectable activity in DMBC19 populations and the highest activity in DMBC17 and DMBC21 populations (Fig 3A). The effect of pentoxifylline on TCF/LEF activity was concentration-dependent (Fig 3B). The highest reduction was observed in the DMBC21 population.

Melanoma cell populations also differed in the expression of β-catenin with a substantially higher level of *CTNNB1* transcript in DMBC21 and DMBC17 populations than DMBC12, DMBC11 and DMBC19 populations (Fig 3C). The β-catenin expression was not markedly changed after treatment with pentoxifylline both on mRNA and protein levels as shown in Fig 3D and 3E, respectively. DMBC17 and DMBC21 populations were then chosen to investigate the effect of pentoxifylline on the level of active form of β-catenin. Western blot analysis indicated that pentoxifylline effectively reduced the level of active, dephosphorylated β-catenin (Fig 3F). Moreover, immunofluorescence microscopic analysis revealed that pentoxifylline substantially reduced nuclear localization of β-catenin in β-catenin^high^ melanoma cells, DMBC17 and DMBC21 (Fig 3G).

### DMBC cell populations differ in basal expression of WNT/β-catenin pathway components

**Fig 4:**
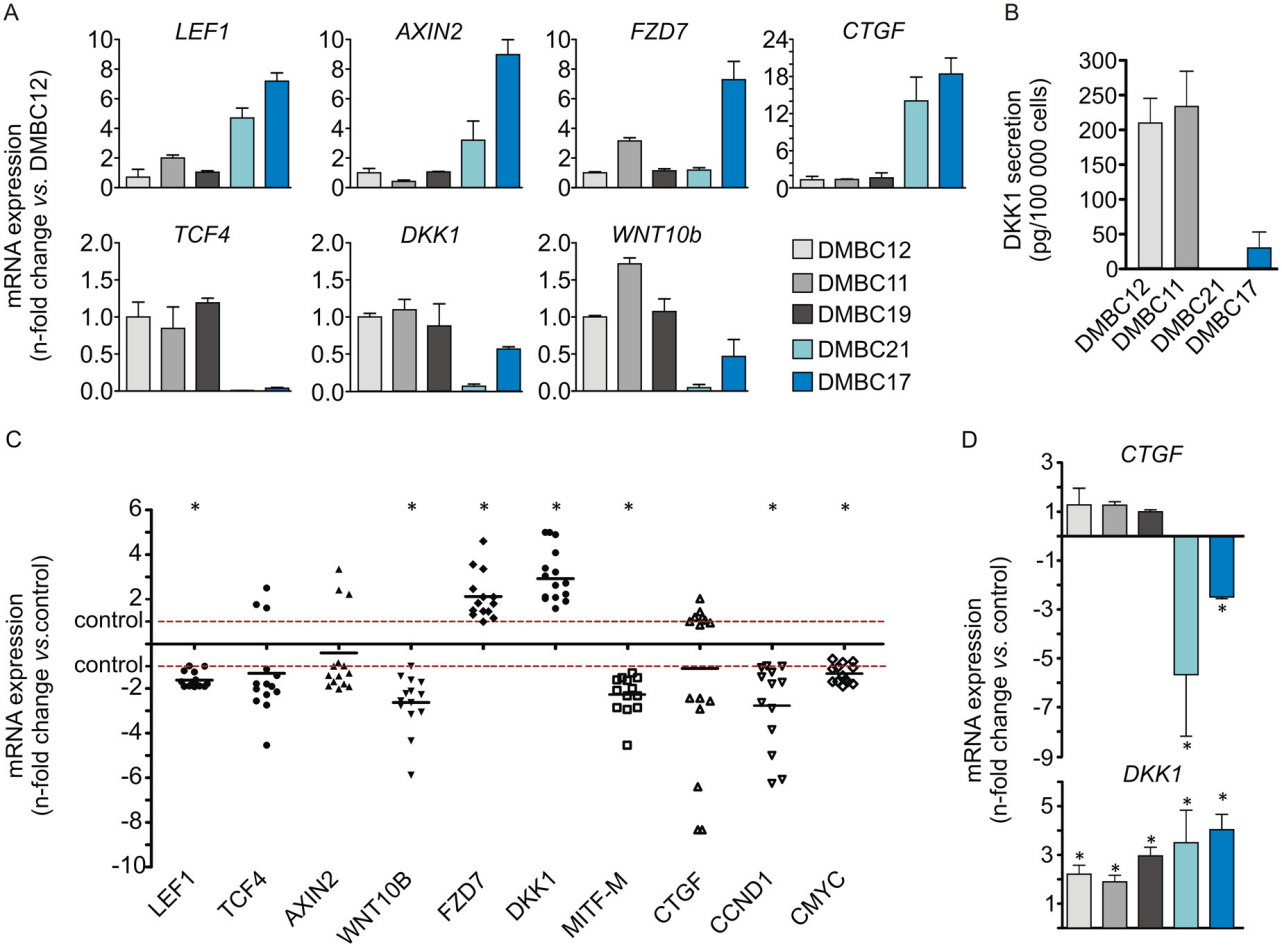
Pentoxifylline (Pen) affects expression of WNT/β-catenin pathway components and target genes. The expression level of each gene was determined by qRT-PCR and normalized to the expression of a reference gene *RPS17*. **(A)** Basal expression levels of WNT/β-catenin pathway components and target genes in each melanoma population relative to their expression in the DMBC12 population. **(B)** DKK1 secretion assessed by ELISA in culture medium collected 24 h after the passage of DMBC11, DMBC12, DMBC21 and DMBC17 populations. **(C)** A fold change in the expression of indicated genes after the treatment with Pen for 18 h relatively to the respective control. Points indicate individual values obtained for each population, and horizontal lines represent means for pooled values. Differences are considered significant at *P < 0.05. **(D)** Pen-induced changes in transcript levels of CTGF and DKK1 shown separately for each melanoma cell population. Differences are considered significant at *P < 0.05.

Next, we determined the basal transcript levels of selected WNT/β-catenin pathway components (Fig 4A). Besides β-catenin co-factors, TCF4 and LEF1, two other WNT/β-catenin pathway components, FXD7 and WNT10B were selected as in the microarray analysis of melanoma transcriptomes, their expression in DMBC cell populations was found to be sensitive to small changes in the microenvironment.^27^ Two inhibitors of WNT/β-catenin pathway, DKK1 and AXIN2, were also included. Interestingly, the gene expression reciprocity between two β-catenin co-factors, TCF4 and LEF1 was observed (Fig 4A). Moreover, the basal expression of *LEF1* was high in the populations with high basal level of β-catenin. Regarding expression of the WNT pathway inhibitors, the transcript of *DKK1* was present at high level in TCF4^high^/β-catenin^low^ cell populations, whereas *AXIN2* was expressed at high level in LEF1^high^/β-catenin^high^ cell populations (Fig 4A). The expression pattern of *WNT10B* was similar to the pattern of *TCF4* and *DKK1* expression. Differences in the *DKK1* transcript levels between DMBC populations were in agreement with differences observed in the secretion level of this inhibitor into the culture medium (Fig 4B).

### Pentoxifylline influences expression of WNT/β-catenin pathway components and target genes

Pentoxifylline induced changes in expression of WNT/β-catenin pathway components and target genes (Fig 4C and 4D). It significantly reduced the transcript levels of *LEF1*, *WNT10B*, *MITF-M*, *c-MYC* and *CCND1* and increased the mRNA levels of *FZD7* and *DKK1* in all melanoma cell populations (Fig 4C). Changes in the expression of other genes such as *TCF4*, *AXIN2* and *CTGF* were more cell population-dependent, and therefore not significant when results for all populations were combined. For instance, the expression of *CTGF* was selectively reduced by pentoxifylline in CTGF^high^/β-catenin^high^ populations, DMBC17 and DMBC21 in contrast to expression of DKK1, which was increased in all melanoma cell populations (Fig 4D).

### Pentoxifylline decreases MITF-M level in melanoma cells and reduces the frequency of MITF/Melan-A-positive cells

**Fig 5:**
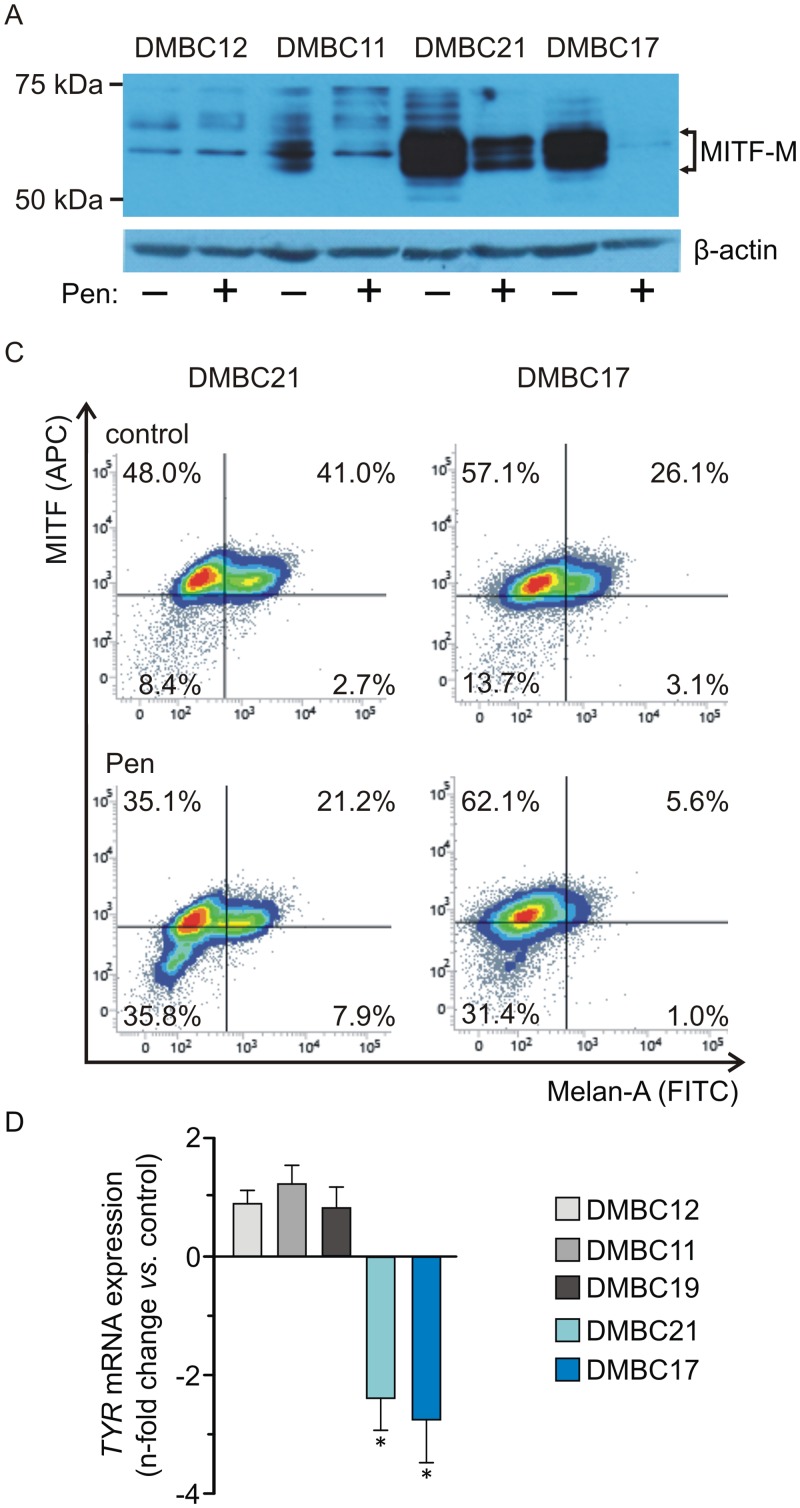
Pentoxifylline (Pen) reduces MITF-M level and its activity and diminishes the subpopulation of MITF^+^/Melan-A^+^ cells. **(A)** Western blot showing changes in MITF-M levels. Cell lysates were prepared after the treatment with 10 mM Pen for 24 h. MITF-M isoform is indicated by arrows. β-actin was used as a loading control. **(B)** Representative flow cytometry density plots showing changes in the percentages of Melan-A- and MITF-positive cells in MITF-M^high^ melanoma cell populations DMBC21 and DMBC17 after the treatment with 10 mM Pen for 24 h. Dead cells were excluded from the analysis using the LIVE/DEAD^®^ Fixable Aqua Dead Cell Stain Kit. **(C)** Pen reduces the transcript level of *TYR*, a MITF-M-dependent gene, after 18 h of treatment. The expression level of *TYR* was normalized to the expression of a reference gene *RPS17*. Data are presented as a fold change in Pen-treated samples *vs*. control. Differences are considered significant at *P < 0.05.

MITF-M is one of the LEF1/β-catenin target genes that is specifically expressed in melanocytes and melanoma cells. Its basal level was very high in DMBC17 and DMBC21 and much lower than in DMBC12, DMBC11 and DMBC19 cell populations (Fig 5A). Western blot analysis revealed that MITF-M migrating as a doublet was markedly reduced by pentoxifylline, especially in MITF-M^high^ populations, DMBC21 and DMBC17 (Fig 5A).

Pentoxifylline substantially reduced the fraction of MITF/Melan-A-positive cells in MITF^high^ cell populations (Fig 5B). It also inhibited the expression of another MITF-M-dependent gene, *TYR* that encodes tyrosinase, an enzyme involved in melanin biosynthesis (Fig 5C).

## Discussion

Despite the recent success of immunotherapies [32–35] and targeted strategies [3,36] that revolutionized melanoma treatment, a subset of patients does not respond to these modalities. Accumulating evidence suggests that the WNT/β-catenin pathway might be one of the determinants responsible for the heterogeneity in therapeutic responses observed across melanoma patients [7,37]. It has been reported that a high level of nuclear β-catenin in biopsies that were taken prior targeted therapy with vemurafenib can be correlated with reduced survival in patients treated with this BRAF^V600E^ inhibitor [37]. Moreover, it has been most recently shown that melanoma-intrinsic active β-catenin signalling prevents anti-tumour immunity by excluding T-cell infiltration into the tumour microenvironment [7]. Therefore, developing drugs that could block β-catenin activity to enhance the potential of immune-mediated melanoma treatment for patients lacking a T-cell infiltrate would be essential.

A key finding of this study is that the activity of the WNT/β-catenin pathway is downregulated by pentoxifylline in melanoma cell populations with high basal activity of this signalling pathway. This conclusion is supported by (i) TCF/LEF reporter assay, (ii) changes in the nuclear localisation of active β-catenin and (iii) reduced expression of WNT/β-catenin pathway components and target genes, including MITF-M and CTGF.

The WNT/β-catenin signalling pathway controls numerous cellular processes and disturbances in this pathway can lead to various diseases, including cancer [24]. Activation of the WNT/β-catenin signalling pathway is tightly regulated and diverse receptors and ligands are involved but a major downstream effector of the canonical WNT pathway is a transcriptional complex of β-catenin with its co-factors TCF4 or LEF1. It has already been shown that the expression of TCF4 and LEF1 is inversely correlated in melanoma, and LEF1 suppresses TCF4 expression in a β-catenin-independent manner [38]. Both β-catenin co-factors, TCF4 and LEF1, were inversely expressed also in the present study. Thus, we had melanoma patient-derived populations with different characteristics, and DMBC17 and DMBC21 cell populations were β-catenin^high^/LEF^high^/TCF^low^ whereas DMBC12, DMBC11 and DMBC19 were β-catenin^low^/LEF^low^/TCF^high^. The translocation of β-catenin to the nucleus is a critical step in the expression of target genes of the WNT/β-catenin pathway [39]. In the present study, pentoxifylline did not substantially affect β-catenin at transcript and protein levels but markedly reduced the level of active β-catenin in the nucleus of melanoma cells with high basal expression of β-catenin. Moreover, changes in the nuclear localisation of active β-catenin were accompanied by reduced expression of two prominent WNT/β-catenin pathway target genes, *c-MYC* and *CCND1*. This reduction was observed in β-catenin^high^ and β-catenin^low^ melanoma cell populations. Decreased cyclin D1 level might contribute to the observed accumulation of melanoma cells in G_0_/G_1_ phase.

Pentoxifylline-induced reduction of other target genes such as *CTGF* and *MITF* seems to be associated with the β-catenin status. The expression of CTGF was reduced by pentoxifylline only in β-catenin^high^/CTGF^high^ cell populations, DMBC21 and DMBC17. CTGF plays diverse roles in normal and pathological states including different cancers, and can be either oncogene or tumour suppressor [40–43]. CTGF is upregulated in melanoma cell lines and primary and metastatic melanoma samples when compared with epidermal melanocytes, and treatment of melanocytes with recombinant CTGF results in the induction of their migratory and invasive phenotype [44]. It has been demonstrated that inhibition of CTGF, either genetically or with a specific monoclonal antibody FG-3019, reduces melanoma growth and prevents the formation of distant metastases in the lungs of SCID mice [45]. Anti-CTGF therapy using monoclonal antibody has entered a clinical trial in patients with locally advanced or metastatic pancreatic ductal adenocarcinoma (NCT01181245.). Our results indicate that CTGF can be also downregulated pharmacologically by a small molecule pentoxifylline in β-catenin^high^/CTGF^high^ melanoma cells. Our study also clearly demonstrates that pentoxifylline inhibits MITF-M expression and activity. MITF-M is a melanocyte-specific transcription factor that has a critical role in the pathogenesis of melanoma [46–49]. The close relationship between the WNT/β-catenin signalling and MITF expression and stability has already been demonstrated [29,50,51]. It has been shown that LEF1 but not TCF4 activates MITF-M expression [38]. This is confirmed in the present study as MITF-M was upregulated only in β-catenin^high^/LEF1^high^ melanoma cell populations, DMBC21 and DMBC17. Pentoxifylline not only efficiently reduced MITF-M protein level, but also significantly downregulated the expression of *TYR*, a MITF-M-dependent gene. Moreover, pentoxifylline diminished the percentage of MITF/Melan-A-positive cells. As MITF is listed among attractive therapeutic targets in melanoma [36], the pentoxifylline-driven downregulation of its expression and activity might have important clinical implications.

Another interesting aspect of our study is the influence of pentoxifylline on expression of *DKK1*, a secreted inhibitor of the WNT/β-catenin pathway. It has been demonstrated that DKK1 expression is reduced in melanoma cells compared with melanocytes [52], but its role in melanomagenesis is not well recognized. Here, we demonstrate that the DKK1 level can be increased by pentoxifylline, and this drug-induced overexpression might be one of mechanisms of WNT/β-catenin pathway downregulation.

## Conclusions

Altogether, it is conceivable that pentoxifylline downregulates the activity of the WNT/β-catenin pathway on multiple levels. High activity of the WNT/β-catenin pathway is associated with decreased patient survival in numerous cancer types [8]. The role of the WNT/β-catenin pathway in melanoma is still unclear. It has been demonstrated that a loss of β-catenin is linked with melanoma progression and activation of the WNT/β-catenin pathway, which is associated with decreased proliferation and suppressed invasion, might be beneficial for melanoma patients [11,12,53–55]. On the contrary, other studies indicate that β-catenin activity increases during melanoma progression, and the WNT/β-catenin pathway promotes melanoma proliferation, cell survival, drug resistance and invasion but not migration of melanoma cells [56,57]. The study of Gallagher et al., suggests that the role of the WNT/β-catenin pathway may be context dependent [57]. Most interesting, as activation of β-catenin pathway in melanoma cells can exclude immune cell activation leading to a non-T cell-inflamed microenvironment, melanoma-intrinsic β-catenin activation is considered as one of the mechanisms of primary resistance to immunotherapies [7]. Since pentoxifylline substantially reduces the β-catenin activity in melanoma cells, this drug might be useful in induction of the melanoma response to CTLA-4, PD-L1 and PD-1 blockade in a subset of patients with the WNT/ β-catenin pathway-driven exclusion of the host immune response. Our *in vitro* data might have important clinical implications and can be facilely verified in clinics as pentoxifylline was shown to be safe for humans and it is approved by FDA to treat patients with chronic peripheral arterial disease. Moreover, preventive effects of a combined regimen of pentoxifylline with other drugs on focal radiation-induced liver injury were observed indicating that pentoxifylline can exert rather protective not damaging effects on normal tissue (ClinicalTrials.gov-identifier NCT01149304) [58].
